# Attitudes of medical students towards communication skills and patient-centered care: the impact of group mentorship

**DOI:** 10.5116/ijme.679e.091b

**Published:** 2025-03-13

**Authors:** Elise Pauline Skjevik, Tor Anvik, Unni Ringberg, Eirik H. Ofstad

**Affiliations:** 1 Department of Community Medicine, UiT The Arctic University of Norway, Tromsø, Norway; 2 Nordland Hospital Trust, Bodø, Norway and Department of Clinical Medicine, UiT The Arctic University of Norway, Tromsø, Norway

**Keywords:** Mentorship, medical education, medical student, professionalism, patient-centeredness

## Abstract

**Objectives:**

To explore medical students’ self-assessed preparedness for clinical practice and attitudes towards learning communication skills, and attitudes towards patient-centeredness before and after introducing a new curriculum with a group mentorship program.

**Methods:**

A cross-sectional questionnaire-study (1-5 Likert scale) was conducted among the first class of medical students following the new curriculum (NC, n = 51) in their fifth year and the final class of students in the old curriculum (OC, n = 48) in their sixth year. The questionnaire contained questions regarding program evaluation, and statements that measured the students’ attitudes towards learning communication skills and patient-centeredness. Descriptive statistics and Mann-Whitney U-test were used.

**Results:**

NC-students (Mdn=4) scored significantly higher than the OC-students (Mdn=3), when asked how they thought the first four years of the medical curriculum had prepared them for clinical practice (U=828.5, p=.003, r=0.35). Similarly, NC-students felt more prepared for communication with patients (Mdn=4 for both groups, U=748.5, p<.001, r=0.35) and ethical reflections (Mdn=4 for both groups, U=951.5, p=0.043, r=0.20). NC-students reported significantly more positive attitudes towards learning communication skills than did OC-students. They had higher mean scores on all items regarding patient-centeredness, although these differences were not statistically significant.

**Conclusions:**

A group-based mentorship program within the new curriculum significantly enhanced medical students’ self-assessed clinical preparedness and positively shifted their attitudes towards communication skills and patient-centeredness. More research is needed to compare medical schools with and without longitudinal group mentorship programs to assess students’ professional attitudes, and ideally, their performance in clinical practice.

## Introduction

Clinical communication is a core skill that is essential for providing correct diagnostic evaluation and treatment, for symptom resolution, and for patients’ satisfaction.^1^^-^^4^ Studies have revealed that teaching communication skills to medical students can have positive effects on empathy, taking medical histories, and interpersonal communication in medical consultations.^5^ Early introduction of communication skills training that runs longitudinally throughout the medical curriculum, has been shown to be effective in improving the students’ psychosocial skills and confidence in clinical settings. ^6^^-^^9^

Communication skills training that includes personalized feedback seems to have the strongest positive impact on medical students’ skills.^5^^,^^10^ Role-playing with simulated patients or peers in small groups is the most common pedagogical method, while observing senior physicians or other students as they interview patients is the most common methods in clinical settings.^5^^,^^11^ Patient-centered approaches aim to ensure efficient communication and shared decision-making, by empowering patients to take a more active role in their care.^12^ This is increasingly recognized as an important topic in health care education, as it may have a positive influence on patients’ health and treatment compliance.^13^^, ^^14^

Nevertheless, some studies have suggested that medical students’ attitudes towards learning and using skills in clinical communication, patient-centeredness, and empathy may decline as they progress through medical school.^15^^-^^18^ This adverse trend has partially been assigned to the effect of the “hidden curriculum”, that is, the informal processes within a curriculum that are often taught unintentionally.^19^^-^^21^ Another theory is that certain methods of communication skills training, such as role-playing with simulated patients in front of peers and teachers, may be a source of stress and anxiety that eventually leads to self-doubt and negative attitudes.^22^

One way to integrate communication skills training and patient-centeredness in medical education, and to mitigate declining empathy among students, is to establish mentorship programs. ^7^^, ^^23^^-^^25^ Many mentorship programs aim to offer support and stimulate professional development, and they focus on empathy, collaboration, ethical decision-making, and patient-centered approaches.^26^^,^^27^ At the medical school at the UiT The Arctic University of Norway (UiT), a new mentorship program was established in 2012, as part of a curricular reform. The overall goal of the revised medical curriculum is to educate physicians with a holistic academic and professional competence that will enable them to treat illness and promote health through patient-centered work.^28^ The new curriculum aimed to use more problem-based learning involving early patient contact and practical training in both general practice and hospital settings, and it implemented a longitudinal group-based mentorship program.

Mentoring in a group-based format can provide rich opportunities for medical students to reflect on social and relational abilities and share experiences with their peers and mentors, resulting in professional development.^23^^, ^^29^ It has been shown that in a group environment, if students can actively compare and build on their own experience alongside their peers, their understanding of knowledge can be enhanced.^30^

To the best of our knowledge, there is little evidence regarding how mentorship programs influence medical students’ attitudes towards professional attributes, especially when compared to students who are not offered such mentoring throughout medical school. The aim of this study was to explore whether the group-based mentorship program within the new curriculum could significantly enhance medical students’ self-assessed clinical preparedness and shift their attitudes towards communication skills and patient-centeredness in a positive direction.

## Methods

### Study context

Medical education in Norway takes six years. Medical students at UiT spend their fifth year away from campus, training in clinical practice under supervision with real patients at small hospitals and in general practice settings. The new curriculum (NC) was introduced for first year students in 2012 and had been implemented for students in their first through fourth years by the summer of 2016. One of the main changes from the old curriculum (OC) was the introduction of a professionalism program (PROFCOM) including a mentorship program that runs longitudinally starting in the first year. Important objectives for “PROFCOM” are learning communication skills and ethics, understanding the physician’s role and professional behavior, and collaboration with other health care professionals. The mentorship groups are an important arena for experiential learning during the first four years and in the sixth year in PROFCOM.

At the time of the study, each year-class of 110 students was divided into 14 groups with two mentors each. The groups consisted of seven to nine students who met with two mentors four times each year, that is, for 16 hours per year. Group meetings are mandatory for the students, and a 75% attendance rate was required. Currently, the attendance rate is 100% with some exceptions. It is also mandatory for the students to meet with one of the mentors for individual feedback and guidance each year.

Each group meeting has one or more predetermined topics or activities. Students bring video-recordings of their own consultations with real patients, or they have consultations with simulated patients in the group, and the group provides feedback. In some meetings, the students write a reflective paper followed by ethical discussions in the groups. See Table 1 for an outline of topics and activities in the mentor groups in years 1-4. Mentors are physicians by training and have a formal affiliation with the University and/or the University Hospital. They do not receive additional financial compensation for being mentors. A half-day orientation seminar allows mentors to meet and prepare for mentoring. Once a year, all mentors are invited to attend one-day follow-up seminars.

### Study design and participants

The present study was part of a larger cross-sectional survey at the UiT The Arctic University of Norway that evaluates the medical curriculum and medical students’ attitudes towards communication and patient-centeredness. We developed a questionnaire containing 15 questions for program evaluation, and 27 statements that measured the students’ attitudes towards learning communication skills and towards patient-centeredness. In a Likert scale ranging from 1 to 5, the items were named “strongly disagree,” “disagree,” “neutral,” “agree,” and “strongly agree.”

**Table 1:** Topics and activities in the mentorship groups at the time of the study

Year/term		Topics*	Activities
Year 1	Autumn	Patients’ experience of living with a chronic illness; what is a good doctor	Video of interview with a patient in general practice
	Spring	How to provide information to patients;uncertainty	Video of role play between students
		Conversation with a patient with breast cancer and her family member; ethics; students' reaction to serious illness	Video of role play between students
Year 2	Autumn	Motivational interview: changing lifestyle; patient autonomy	Video of role play between students
		Gathering information from a patient; integrating information about current medical problem; the patient’s perspective; communication skills	Video of interview with a patient at either an outpatient or inpatient clinic
	Spring	“Ethics in everyday medical practice”; a patient encounter that affected the student emotionally	Students write a reflective paper for discussion about ethics in groups
		History-taking in general practice	Video of patient encounter in general practice
Year 3	Autumn	Gathering information from a patient; integrating information about a current medical problem; the patient’s perspective; communication skills	Video of interview with a patient at either an outpatient or inpatient clinic
		Same as above	Same as above
	Spring	History-taking; examination; analysis and planning with a patient in general practice	Video of a patient encounter in general practice
		“Ethics in everyday medical practice”; a patient encounter where a physician behaved in an unfortunate manner	Students write a reflective paper for a discussion about ethics in groups
Year 4	Autumn	History-taking with a pregnant woman referred for an early ultrasound; ethical topics in gynecology and obstetrics	Video with a pregnant woman and her partner in a gynecological outpatient clinic; discussion and reflection of ethical topics
		Routine control of children at healthcare centers, history-taking, and examination; clinical encounters with children; providing “bad news” to parents of a newborn with Downs syndrome	Video with children and their relatives at the healthcare center; videos on YouTube; role-playing in the mentor groups with simulated patients (the parents)
	Spring	Ambivalence, abortion; physicians on night shifts; patients’ experience of how to live with a chronic, potentially lethal disease and GPs’ experience of providing health care (after lecture with a patient)	Role-playing in the group with a simulated patient and ethical reflections; reflection notes and discussions in the group after shadowing a physician during a night shift at the hospital; reflection notes and discussions.
		History-taking; examinations; analysis and planning of patients in general practice	Consultations with simulated patients in the group

*In most meetings, there is also time to discuss topics that students raise about professionalism, communication, and ethics

**Figure 1:**
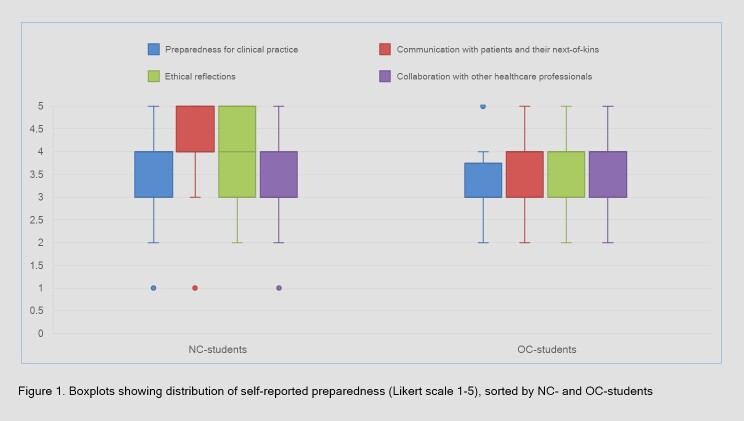
Boxplots showing distribution of self-reported preparedness (Likert scale 1-5), sorted by NC- and OC-students

**Figure 2:**
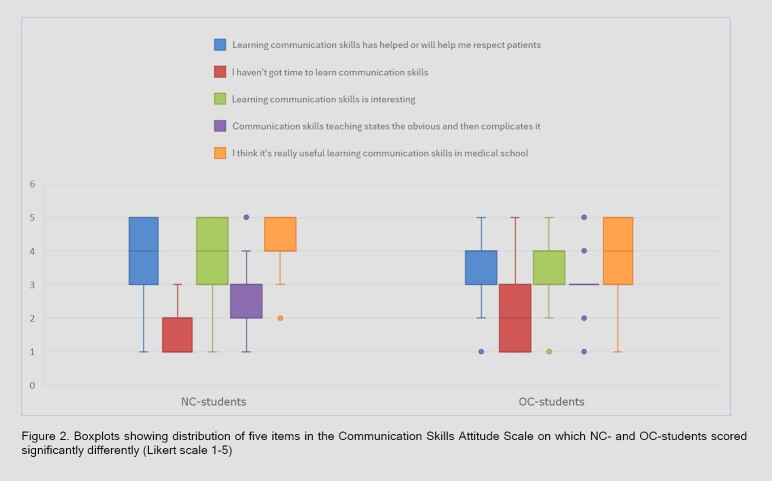
Boxplots showing distribution of five items in the Communication Skills Attitude Scale on which NC- and OC-students scored significantly differently (Likert scale 1-5)

The 15 questions regarding program evaluation asked the students to score the extent to which they felt that the curriculum in general and specific teaching sessions in particular had prepared them for clinical practice. The 27 questions regarding attitudes were based on two validated tools. The first was the Norwegian version of the Communication Skills Attitudes Scale (CSAS).^31^^,^^32^ This version comprises 22 statements regarding attitudes towards learning communication skills. Ten of the statements are negatively worded (e.g., “I can’t see the point in learning communication skills”), and 12 statements are positively worded (e.g., “Learning communication skills is interesting”). The statements are presented in a random order.^32^ Both the positively and negatively worded statements exceeded an alpha value above 0.8, indicating an acceptable internal consistency. Further, a satisfactory test-retest reliability using the kappa coefficients was found.^33^

The second tool was based on a survey analyzing medical students’ attitudes towards patient-centered versus physician-centered practice.^34^ The original study tested a total of 17 statements and found that five of these specifically measured patient-centered attitudes (e.g.: “The physician should clarify with the patient what they will discuss in the consultation”). We used these five statements to measure patient-centeredness. This tool yielded a Cronbach alpha of 0.461 (items 10, 12, and 13) and 0.626 (items 5 and 8).^34^

The students were invited to participate by e-mail. At the time of the study, the NC-students had finished their fifth year of medical school, when they were deployed to hospitals and general practice offices throughout northern Norway. The OC-students were in their sixth and final year. After piloting and adapting the survey to Questback, an invitation was sent by e-mail to the eligible NC-students (n=88, 71.6% women) and the 90 OC-students (n=90, 62.2% women) at UiT in the Spring of 2017. The Norwegian Centre for Research Data approved the study. All participants volunteered, and strict measures were in place to ensure the anonymity of all respondents. A total of 51 (74.5% female) NC-students and 48 (58.3% female) OC-students responded. Table 2 presents the characteristics of the invited and responding NC- and OC-students.

### Data analysis

The statistical analyses were performed by authors EPS and UR in SPSS version 29. Descriptive statistics were conducted for the participants’ characteristics. The Mann-Whitney U-test for two independent samples was used to explore the differences between NC- and OC-students regarding curriculum evaluation and the students’ attitudes towards teaching communication skills and patient-centeredness. The effect sizes (r) were measured by dividing the standardized test statistic (z) by the square root of the number of observations. According to Cohen's categorization of effect sizes, 0.1 is considered small, 0.3 represents a moderate effect, and values of 0.5 or higher imply a large effect. 35 The significance threshold for the analyses was set at 5% (p < 0.05).

## Results

In the following comparisons between NC- and the OC-students, the results from the Mann Whitney U-test are reported. The NC-students (Mdn=4) scored significantly higher than the OC-students (Mdn=3), when asked how they thought the first four years of the medical curriculum had prepared them for clinical practice (Table 3) (U=828.5, p=.003, r=0.35).

Additionally, the NC-students scored significantly higher than the OC-students when asked how the curriculum had prepared them for communicating with patients (Mdn=4 for both groups, U=748.5, p<.001, r=0.35), and on how the curriculum had prepared them for ethical reflections in clinical practice (Mdn=4 for both groups, U=951.5, p=.043, r=0.20). The OC-students (Mdn=4) felt more prepared to collaborate with other health care professionals than the NC-students (Mdn=3), but this finding was not significant (U=1297.5, p=.584, r=0.05). Figure 1 provides boxplots visualizing the NC- and the OC-students’ responses to these items.

The NC-students were asked how the mentorship groups and PROFCOM had prepared them for clinical practice. The median scores were 4 (IQR = 1) for both items. Further, these students were asked about the extent to which they thought each of the 10 mentorship activities had prepared them for clinical practice. Most activities had a median score of 3, and the lowest median score was 2 (Table 4).

When analyzed individually, the scores on five of the 22 items in the CSAS differed significantly between the NC- and OC-students. The NC-students scored significantly higher on three items that were positively worded and lower on two that were negatively worded (Table 5). Figure 2 visualizes the responses of NC- and OC-students on these five items. On the items regarding patient-centeredness, the NC-students had higher scores on all five statements, though none of these differences were statistically significant (Table 6).

## Discussions

One crucial test for the relevance and efficiency of a teaching program for medical students is whether students feel that the program is helpful in preparing them for working with real patients in everyday clinical settings. Students at UiT spend most of their fifth year training with real patients in small hospitals and in general practice, giving them abundant opportunities to experience how they personally feel prepared for real life medical practice. In this study, we found that the first cohort of students enrolled in the new curriculum (NC-students) felt better prepared for clinical practice during their fifth year than did the OC-students. Specifically, the NC-students felt more prepared for ethical reflections and for communicating with patients in clinical practice.

**Table 2:** NC- and OC-students’ characteristics

Students	Invited						Respondents						
	Female		Male		Sum		Female		Male		Sum		
	n	%	n	%	n	%	n	%	n	%	n	%	
NC	63	71.6	25	28.4	88*	100	38	74.5	13	25.5	51*	100	
% of invited							60.3		52		57.9		
OC	56	62.2	34	37.8	90	100	28	58.3	20	41.7	48	100	
% of invited							50.0		58.8		53.3		
													

*Eight students were excluded from the analyses as they reported that they did not attend any mentorship activities

Note: NC: The first class of students enrolled in the new curriculum; in their fifth year at the time of the study. OC: The final class of students using the old curriculum; in their sixth and final year at the time of the study

**Table 3:** NC-students’ assessment of how the mentorship groups prepared them for clinical practice

Item		Students	Median*	U	Z	Effect size (r)	p-value
How the first four years of medical curriculum prepared them for clinical practice		NC	4	828.5	-3.58	0.35	.003**
		OC	3				
How the medical curriculum prepared them for:	communication with patients and their next-of-kin	NC	4	748.5	-3.58	0.35	< .001**
		OC	4				
	ethical reflections	NC	4	951.5	-2.01	0.20	.043**
		OC	4				
	collaboration with other health care professionals	NC	3	1297.5	.55	0.05	.584
		OC	4				

*Likert score 1–5

**Statistically significant, p<0.05

**Table 4:** NC-students’ assessment of how the mentorship groups prepared them for clinical practice

Survey question	Activities	Median Likert*score (IQR)**
To what extent do you think that the mentorship groups have prepared you for clinical practice?		4 (1)
To what extent do you think that PROFCOM has prepared you for clinical practice?		4 (1)
To what extent do you think that each of these activities in the mentorship groups has prepared you for clinical practice?	Feedback on video with a patient in general practice	3 (1)
	Feedback on video with a patient in an outpatient clinic or bed ward	3 (1)
	Feedback on video with a patient at the health center	3 (1)
	Feedback on video of role play with a peer student	2 (1)
	“Consultation” with a simulated patient in the groups	3 (1.25)
	Reflection notes after shadowing a physician during a night shift in the hospital, followed by discussion in groups	2 (1)
	Reflection notes on ethical challenges, followed by discussion in groups	3 (2)
	Discussions on YouTube videos in groups	3 (1.75)
	Individual talk with one of the mentors	3 (2)

*Likert scale ranged from 1 – to a very small extent to 5 – to a very large extent

**IQR = Interquartile range, Q3-Q1

**Table 5:** Items in the Communication Skills Attitude Scale on which NC- and OC-students scored significantly differently

No.	Item	NC students	OC students	U	Z	Effect size (r)	p-value*
		Median	Median				
5	Learning communication skills has helped or will help me respect patients	4	4	1032	-2.51	0.25	.012
6	I haven't got time to learn communication skills	1	2	848	3.99	0.40	< .001
7	Learning communication skills is interesting	4	4	1101	-2.07	0.20	.039
11	Communication skills teaching states the obvious and then complicates it	3	3	1097.5	2.13	0.21	.033
18	I think it's really useful learning communication skills in medical school	4	4	1055.5	-2.39	0.24	.017

*p<0.05, Mann-Whitney U-test

**Table 6:** Median scores on items regarding patient-centered attitudes* (NC and OC students)

No.	Item	NC-students	OC-students	U	Z	Effect size (r)	p-value**
		Median	Median				
5	The patient should express agreement with the physician to signal respect and trust	2	1.5	1219.5	-1.29	0.13	.195
8	The patient should relate to what the physician says and not seek information about their illness on their own	2	2	1350.5	-0.43	0.04	.664
10	The physician should consider the patient’s advice in medical decision-making	5	5	1317	-0.80	0.08	.421
12	The patient's description of the symptoms is important to get the correct diagnosis	5	5	1276	-1.20	0.12	.233
13	The patient should be treated as the physician’s equal, equivalent in power and status	4	4	1250	-1.10	0.11	.272

*The five items that specifically measure patient-centered attitudes in the study by Solheim & colleagues.

**Mann-Whitney U-test

They also expressed positivity towards the mentorship groups and the professionalism program that they were a part of, and felt that that it had adequately prepared them for clinical practice. Another significant finding was that the NC-students reported more positive attitudes towards learning clinical communication skills. Additionally, they scored higher on all survey questions related to patient-centeredness than the OC-students, although these differences were not statistically significant.

Surprisingly, we found a median score of 3 among NC-students for several of the specific mentorship group activities, even though they responded positively about how the program in general had prepared them for clinical practice. Most of these activities involved recording and watching videos of role-playing or students’ own encounters with simulated or real patients. Possible reasons for this discrepancy include students feeling uncomfortable and lacking experience in role-playing and video recording, and in receiving and giving structured feedback on communication skills in group settings. The use of video-cameras can generate technical challenges, and if relied on repeatedly, this can lead to frustration. Furthermore, experiential learning, such as patient interviews that peer students and mentors observe, has been shown to cause stress, tension, and feelings of embarrassment.^22^^, ^^36^ This could be especially prominent in inexperienced students, that is, those just starting medical school.^37^

Despite the relatively low scores on several of the mentorship activities, the NC-students reported a high median score on the question regarding how the mentorship groups in general had prepared them for clinical practice. They scored the mentorship groups slightly higher than the overall curriculum regarding their preparedness for clinical practice. One explanation for this is the potential positive effect of the “hidden curriculum”, in that role- and behavior modelling can transmit values that are important in clinical practice, such as ethical thinking, responsibility and patient-centeredness.^38^^,^^39^ Previous studies have shown that well-functioning group mentorships can be an effective way to provide students with beneficial role models to learn from and emulate, and can allow them to evolve professionally in the company of peer students.^23^^,^^40^ Both mentors and peer students may act as important role models in these settings. ^41^

Another interesting finding in our study was that both the NC- and the OC-students rated every positively worded CSAS items relatively high. At the time of the study, the NC-students were in their fifth year and the OC-students were in the sixth and final year. Other studies have reported that medical students tend to develop more negative attitudes towards patient-centeredness and communication as they progress through medical school,^15^^,^^17^ so one may expect that the attitudes to be negatively skewed. However, a Norwegian study at two universities showed that medical students’ attitudes towards learning communication skills at the end of medical school had improved over a 12-year period.

The authors suggest that this may illustrate the increasing expectation for physicians to have higher levels of communication skills, hence leading to greater motivation among the students.^9^

Attitudes motivate behavior, and positive attitudes are well known to contribute to obtaining specific skills. ^42^ In the same way, experiencing unprofessional behavior and poor role modelling can have a strong impact on students’ attitudes and further behavior.^43^^, ^^44^ Overall, the NC-students had more positive attitudes towards learning communication skills and towards patient-centeredness. They highly rated the items stating that learning communication skills is interesting, and that it has helped or will help them respect their patients. This finding aligns well with previous knowledge, as it has been shown that discussions in small groups and constructive feedback on students’ patient encounters were associated with improvement in student performance, compared to other teaching approaches (e.g. lectures).^45^

Previous studies have reported that longitudinal and integrated training in medical school can improve psychosocial skills, such as communication skills and empathy.^9^^,^^24^^,^^46^ Participating in reflective discussions with peers, particularly if accompanied by positive role models, helps students in developing psychosocial skills.^47^^,^^48^ Based on existing knowledge, this study proposes that a decline in attitudes may not necessarily be solely attributed to changes in students’ cognitive attitudes. Poor learning experiences may also contribute to the development of less positive attitudes.^25^^,^^32^ The consistently positive attitudes among NC-students towards communication skills and patient-centeredness calls for a deeper exploration of the factors that influence positive learning environments in medical education.

The findings of this study offer insights into the potential of a group-based mentorship program in medical education to foster positive attitudes towards important interpersonal skills. Hopefully, our findings can highlight the importance of incorporating elements that specifically prepare students for real-world clinical practice, patient communication, and ethical decision-making to better equip future physicians for the complexities of clinical practice.

### Limitations

A possible limitation of this study is that the students responding to our survey may have had more positive feelings towards the curriculum, the teaching of communication skills teaching and patient-centeredness than the non-responding students. Furthermore, it is likely that the first class of mentors at UiT were highly motivated, which may have affected the NC-students’ positive assessment of the mentorship program. Therefore, it is essential to repeat this evaluation. In any study that measures respondents’ attitudes, the possibility of response bias exists. This occurs when participants provide inaccurate answers to questions, and bias can occur if they choose to report what they believe is socially acceptable.^49^

Another limitation concerns the possibility of recall bias, when time affects memory.^50^ It may have been challenging for the NC-students to recall how they experienced each of the mentorship activities in the beginning of medical school. Further, this study was conducted at a single university, which limits generalizability, but our results may be relevant for other universities that educate doctors. The cross-sectional design of this study makes it challenging to infer causality.^51^

Future research should compare medical schools with and without longitudinal group mentorship programs with regards to both the students’ attitudes towards communication skills training and patient-centeredness, and ideally how they perform in clinical practice. To mitigate recall bias, it may be beneficial to use shorter recall periods or conducting mixed methods studies. Further research with a larger sample size could explore the significance of the differences observed.

## Conclusions

Medical students who followed a longitudinal group-based mentorship program felt better prepared for clinical practice than students in a traditional curriculum. The findings also revealed a positively shift in the students’ attitudes towards communication skills and patient-centeredness. This indicates that group-based mentorships can be a valuable teaching resource. Hopefully, our findings can highlight the importance of incorporating elements that specifically prepare future physicians for clinical practice, patient communication, and ethical decision-making. More research is needed to further explore influences on students’ professional attitudes, and how students with and without a longitudinal group mentorship perform in clinical practice.

### Conflict of Interest

The author declares that there is no conflict of interest.
